# Pre-cART Immune Parameters in People Living With HIV Might Help Predict CD8+ T-Cell Characteristics, Inflammation Levels, and Reservoir Composition After Effective cART

**DOI:** 10.20411/pai.v6i2.447

**Published:** 2021-10-01

**Authors:** Jimena Salido, Alejandro Czernikier, César Trifone, María Laura Polo, María Inés Figueroa, Alejandra Urioste, Pedro Cahn, Omar Sued, Horacio Salomon, Natalia Laufer, Yanina Ghiglione, Gabriela Turk

**Affiliations:** 1 Universidad de Buenos Aires, Facultad de Medicina, Departamento de Microbiología, Parasitología e Inmunología, Buenos Aires, Argentina; 2 CONICET – Universidad de Buenos Aires, Instituto de Investigaciones Biomédicas en Retrovirus y SIDA (INBIRS), Buenos Aires, Argentina; 3 Universidad de Buenos Aires, Facultad de Medicina, Buenos Aires, Argentina; 4 Fundación Huésped, Buenos Aires, Argentina; 5 Hospital General de Agudos “Dr. JA Fernández” Buenos Aires, Argentina

**Keywords:** HIV, persistence, CD8 T cells, CD4 T cells, inflammation, viral reservoir

## Abstract

**Background:**

Combined antiretroviral treatment (cART) for HIV infection is highly effective in controlling viral replication. However, it cannot achieve a sterilizing cure. Several strategies have been proposed to achieve a functional cure, some of them based on immune-mediated clearing of persistently infected cells. Here, we aimed at identifying factors related to CD8TC and CD4TC quality before cART initiation that associate with the persistence of CD8TC antiviral response after cART, inflammation levels, and the size of the viral reservoir.

**Methods:**

Samples from 25 persons living with HIV were obtained before and after (15 months) cART initiation. Phenotype and functionality of bulk and HIV-specific T cells were assayed by flow cytometry *ex vivo* or after expansion in pre-cART or post-cART samples, respectively. Cell-Associated (CA) HIV DNA (total and integrated) and RNA (unspliced [US] and multiple spliced [MS]) were quantitated by real-time PCR on post-cART samples. Post-cART plasma levels of CXCL10 (IP-10), soluble CD14 (sCD14) and soluble CD163 (sCD163) were measured by ELISA.

**Results:**

Pre-cART phenotype of CD8TCs and magnitude and phenotype of HIV-specific response correlated with the phenotype and functionality of CD8TCs post-cART. Moreover, the phenotype of the CD8TCs pre-cART correlated with markers of HIV persistence and inflammation post-cART. Finally, exhaustion and differentiation of CD4TCs pre-cART were associated with the composition of the HIV reservoir post-cART and the level of inflammation.

**Conclusions:**

Overall, this work provides data to help understand and identify parameters that could be used as markers in the development of immune-based functional HIV cure strategies.

## INTRODUCTION

Human Immunodeficiency Virus (HIV) is a retrovirus that causes a chronic infection, which produces an irreversible and profound deterioration of the immune system, ultimately leading to the development of acquired immunodeficiency syndrome (AIDS) in the vast majority of untreated HIV^+^ persons [1]. Combination antiretroviral treatment (cART) has drastically improved the life expectancy and quality of life of people living with HIV/AIDS (PLWHA). Simultaneously, cART has an impact on the epidemic dynamics since good coverage of suppressive cART has been proven to lower transmission rates. Finally, cART is available as pre-exposure or post-exposure prophylaxis [2, 3]. Once cART is initiated, viral replication is suppressed and plasma viral load (VL) falls below detectable levels but, if treatment is interrupted, VL rapidly increases as a consequence of viral reactivation from the latent reservoir [4–6]. Consequently, cART alone is not sufficient to eliminate the infection and PLWHA are dependent on a lifelong treatment which still has several limitations in terms of administration, toxicity, and resistance, among others [7, 8]. On the other hand, there are several reports describing cases of PLWHA who had shown long-term control of the infection either spontaneously or induced by an intervention [9–13] indicating that achieving virus control is feasible. Based on this, different strategies have been designed to find a cure for HIV infection [14, 15].

The ability to maintain viral control is multifactorial and involves host features (clinical, genetic, immune, metabolic, etc), as well as viral features (genome integrity, fitness, escape mutations). However, HIV-specific CD8^+^ T cells (CD8TC) emerge as key factors to achieve control in different models (reviewed in [16]). CD8TC specificity, function, phenotype, and localization are determinants of viral control and disease progression and may determine, at least in part, the success of future HIV cure and prevention strategies.

Our group has provided evidence regarding different qualitative aspects of the CD8TC response (specificity, functionality, and phenotype) that better associate with virus control in patients not receiving cART in an acute infection cohort from Argentina [17–19]. In the same cohort, we also determined that a higher proportion of terminally differentiated CD8TC and increased PD1 expression before cART initiation were correlated with HIV persistence once cART was initiated [20]. In another report, we analyzed the phenotype (memory/effector maturation subsets and PD-1 expression) and function (production of IFN-γ, IL-2, CCL4[MIP-1β], and/or TNF-α; CD1071/b mobilization, and direct and indirect antiviral activity) of *in vitro* expanded CD8TC from PLWHA receiving cART who initiated treatment either early or late after infection [21]. Results indicated that HIV-specific cells could be selectively stimulated and expanded *in vitro*, presenting a high proportion of cells able to carry out multiple effector functions. Although cART initiation timing had an impact on the memory/effector differentiation phenotype, expanded cells from both groups had potent antiviral activity. Here, we aimed at extending our previous work by providing a more complete picture that identifies factors related to CD8TC and CD4TC quality before cART initiation that associates with the persistence of CD8TC antiviral response after cART, inflammation levels, and viral reservoir size. For this, distribution of memory sub-populations and PD-1 expression at CD4TC and CD8TC compartments, and polyfunctionality of HIV-specific CD8TCs were evaluated pre-cART. Additionally, the distribution of memory/effector subsets in CD8TC, HIV-specific CD8TC polyfunctionality, cell-associated (CA) HIV DNA and RNA forms, and plasma levels of IP-10, sCD14, and sCD163 were determined in samples obtained post-cART. Then, correlation analyses were performed between pre-cART and post-cART measurements.

## METHODS

### Study subjects:

Twenty-five participants diagnosed with HIV infection were enrolled as part of the *Grupo Argentino de Seroconversión* study group [22] and included in this study. Plasma and peripheral blood mononuclear cells (PBMCs) were collected before cART initiation (pre-cART sample, median time from the presumed date of infection=90 days) and after cART initiation (post-cART sample, median= 15 months on cART) (Table 1). This study was reviewed and approved by the institutional review board *Comité de Ética Humana, Facultad de Medicina, Universidad de Buenos Aires*, Buenos Aires, Argentina. All participants provided written informed consent.

**Table 1:** Characteristics of enrolled participants.

				Pre-ART sample^b^					Post-ART sample^c^				
Subject ID^a^	Gender	Age	ART regimen	Viral Load^d^ (HIV RNA copies/ml plasma)	CD4 count^e^ (cells/ml)	CD8 count^e^ (cells/ml)	CD4/CD8 ratio	Time from infection^f^ (days) (HIV RNA copies/ml plasma)	Viral Load^d^ (cells/ml)	CD4 count^e^ (cells/ml)	CD8 count^e^	CD4/CD8 ratio	Time from ART initi-ation^g^ (days)
**DT1**	F	34	3TC AZT LPV/r	48526	383	531	0,72	1140	<50	562	513	1,09	960
**DT2**	M	34	TDF 3TC ATV/r	132317	324	703	0,46	630	<50	297	571	0,52	210
**DT3**	M	42	3TC ABC EFV	125716	391	602	1,36	210	<50	711	1094	0,65	570
**DT4**	M	25	NA	27244	465	1027	0,45	330	<50	535	723	0,74	150
**DT5**	F	23	NA	NA	NA	NA	NA	NA	<40	739	739	1	210
**DT6**	M	26	3TC AZT EFV	66929	246	1047	0,24	570	<50	501	808	0,62	330
**DT7**	M	45	3TCLPV	15297	330	780	0,42	1530	<50	468	608	0,77	150
**DT8**	F	43	NA	>500000	279	1085	0,26	120	<40	528	733	0,72	270
**DT9**	M	42	NA	28288	184	606	0,3	640	<40	579	657	0,88	450
**DT10**	M	37	3TC AZT EFV	NA	NA	NA	NA	NA	<40	563	780	0,72	600
**DT11**	M	49	3TC AZT EFV	NA	NA	NA	NA	NA	<40	1023	674	1,52	1350
**DT12**	M	27	TDF FTC EFV	111893	629	741	0,85	240	ND	696	472	1,48	690
**Median DT (IQR25%-75%)**				57728 (27505122260)	330 (263428)	741 (6041037)	0,45 (0,280,79)	570(225-890)		563 (508707)	699 (580770)	1 (0,671,07)	390(210 - 668)
**ET1**	M	39	TDF FTC ATV/r	>500000	627	943	0,66	30	<40	594	668	0,89	450
**ET2**	M	45	3TC AZT EFV	283441	187	928	0,2	90	<40	522	596	0,88	600
**ET3**	M	41	TDF ATD 3FTC	36338	768	649	1,18	30	<50	1099	599	1,83	330
**ET4**	M	25	TDF FTC EFV	17292	612	1828	0,33	60	<50	1342	1010	1,31	450
**ET5**	M	52	TDF 3TC ATV/r	20106	787	743	1,06	60	<40	829	491	1,69	480
**ET6**	M	70	TDF FTC	>500000	421	1379	0,31	60	<40	854	452	1,89	540
**ET7**	M	26	TDF FTC EFV	671440	435	3346	0,13	30	<40	842	646	1,3	510
**ET8**	M	30	TDF 3TC ATV/r	172468	345	761	0,45	30	<40	657	566	1,16	360
**ET9**	M	31	TDF FTC EFV	26547	400	571	0,7	30	ND	824	413	1,99	1000
**ET10**	M	35	NA	231360	330	1881	0,18	60	<40	1080	868	1,24	360
**ET11**	M	30	NA	9294958	654	1570	0,42	30	<40	1026	696	1,47	360
**ET12**	M	26	TDF FTC EFV	>500000	339	4308	0,08	90	<40	691	1044	0,66	480
**ET13**	M	42	TDF FTC ATV/r	>500000	213	1126	0,19	90	<40	386	480	0,81	360
**Median ET (IQR25%-75%)**				172468 (23327477441)	421 (335640)	1126 (7521855)	0,33 (0,190,70)	60 (30-75)		829 (6261053)	599 (489782)	1,3 (0,891,76)	450 (360 - 525)
**Median Total (IQR25%-75%)**				66929 (26896201914)	387 (313616)	935 (6891427)	0,42 (0,230,70)	90 (30390)		691 (531848)	657 (539759)	1 (0,731,47)	450 (330 - 585)

a Participants denoted as DT initiated ART after 4 months since the estimated date of infection (DT=Delayed Treatment). Participants denoted as ET initiated ART within 4 months post-infection (ET=Early Treatment). Further analyses were performed using data from all participants without segregation. All study participants were White.

b Sample obtained immediately before participants started ART.

c Sample obtained on ART.

d Versant HIV-1 RNA 3.0 assay (Siemens. Lower and upper detection limits are 50 and 500,000 RNA copies/ml, respectively) or Abbott Real Time HIV-1 assay (Abbott Park, Lower and upper detection limits are 40 and 10^7^ RNA copies/ml, respectively. ND: Non-detected).

e Single platform flow cytometry (BD Biosciences, USA).

f Relative to the presumed date of infection.

g Time from the moment of cART initiation to sample obtaining.

F: Female. M: Male. NA= Data not available.

Drugs = 3TC: Lamivudine; AZT: Zidovudine; LPV/r: Lopinavir/ritonavir; TDF: Tenofovir; ATV/r: Atazanavir/ritonavir; ABC: Abacavir; EFV: Efavirenz; FTC: Emtricitabine

### Samples:

We collected 40 ml of whole blood. Upon centrifugation, plasma was recovered, and stored at −80C. Peripheral blood mononuclear cells (PBMCs) were isolated by Ficoll-Hypaque density gradient centrifugation (Amersham, Sweden) and cryopreserved in liquid nitrogen for subsequent functional assays. Plasma viral load (VL) was determined by branched-DNA, Versant HIV-1 RNA 3.0 assay (Siemens Healthcare, UK) or by Abbott Real Time HIV-1 assay (Abbott Park, IL) depending on kit availability.

CD4+ and CD8+ T-cell counts were determined by flow cytometry (FACSCalibur; BD Biosciences, USA). Cellular immune activation was evaluated as the percentage of CD38- and/or HLA-DR-expressing CD4+ and CD8+ T cells by flow cytometry.

### *Ex vivo* phenotype and functionality of bulk and HIV-specific T-cells by flow cytometry:

Pre-cART samples were thawed, rested for 2 hours, stimulated with HIV peptide pools for 5 hours and then stained as described previously by our group [21]. Peptide pools spanning Nef (127 peptides) or p24 (128 peptides) were constructed using peptides from the potential T-cell epitope (PTE) peptide panels obtained from the NIH AIDS Reagent Program [23]. For stimulation, peptide pools were used at 2 μg/mL together with costimulatory antibodies (anti-CD28 and anti-CD49d; 1 μg/mL; BD Biosciences), monensin (Golgistop, 0.7 μL/mL; BD Biosciences) and brefeldin A (10 μg/mL; BD Biosciences). A DMSO condition was included to account for background. To evaluate polyfunctionality, anti-CD107a/b-FITC antibodies (BD Biosciences) were also added to identify degranulating cells. After stimulation, cells were further stained with Zombie NIR™ Fixable Viability Kit (Biolegend, USA), and the following conjugated antibodies: anti-CD14-V450, anti-CD19-V450, anti-CD3-BV786, anti-CD8-APC, and anti-CD4-BV650 (BD Biosciences). Then, cells were permeabilized (Permeabilization Wash Buffer, Biolegend), fixed (Fixation Buffer, Biolegend), and subsequently stained using anti-IL-2–PerCP-Cy5.5, anti-TNF-α-PECy7, anti-IFN-γ-BV711, and anti-CCL4-PE conjugated antibodies (BD Biosciences).

In parallel, T-cell memory phenotype was studied as also described by our group [21]. Cells were briefly stimulated as described above and afterwards stained with Zombie NIR™ Fixable Viability Kit plus the following conjugated antibodies, anti-CCR7-Alexa700, anti-PD-1-PE, anti-CD3-BV786, anti-CD8-APC, anti-CD4-BV650, anti-CD14-V450, anti-CD19-V450, anti-CD45RO-Per-CPCy5.5, and anti-CD95-PE-CF594 (BD Biosciences, USA). Following permeabilization, cells were fixed and stained with anti-IL-2, anti-TNF-α, and anti-IFN-γ antibodies, all of them conjugated to FITC (BD Biosciences) to identify specific cells regardless of function.

Flow cytometry data acquisition was performed on a 3-laser 14-color BD FACSAria FUSION flow cytometer using the BD FACSDiva v 8.0.1 software (BD Biosciences). Instrument settings and fluorescence compensation were performed using unstained samples and single-stained BD CompBeads (BD Bioscience). Isotype controls, consisting of stimulated cells stained with conjugated antibodies to CD14, CD19, CD3, CD4, and CD8 plus the isotype controls corresponding to the CCR7, CD45RO, CD95, PD-1, and/or the corresponding intracellular marker were performed for each individual in order to set negative populations accurately.

Acquired data was analyzed using FlowJo v10 (Data Analysis Software, LLC). Gating strategy was performed as described before [21]. First, single cells were selected in a forward scatter area (FSC-A) vs FSC-Height plot. Then, dead cells were excluded based on Zombie NIR™ fluorescence, and monocytes and B lymphocytes were also excluded according to CD14 and CD19 staining. Subsequently, the lymphocyte population was selected in an FSC-A vs side scatter (SSC) plot. Samples with at least 100,000 events in the lymphocyte gate were included in subsequent analyses. Finally, CD3+ CD8+ (or CD4+) cells were gated in CD3-vs-CD8 (or CD4) dot plots.

To study T-cell polyfunctionality, CD107a/b, IFN-γ, IL-2, CCL4, or TNF-α plots were constructed on the CD8+ and CD4+ populations and a Boolean gate platform was used to create all possible combinations. Data presented correspond to background-subtracted results using the DMSO plus CD28/CD49d control. This was performed on a cytokine-subset-by-cytokine-subset basis, ie, subtracting the result from this condition for a given cytokine subset to the same subset of a peptide-stimulated condition. One standard deviation (SDs) above background was set as the threshold for determining positive responses. Values below this threshold were set at 0.

The distribution of the different T-cell phenotype subsets was studied in bulk and HIV-specific CD8+ T cells and bulk CD4+ T cells. HIV-specific CD8+ T cells were identified in a CD8 vs FITC plot (CD107a/b, IFN-γ, IL-2, CCL4, and TNF-α). A positive cytokine response was defined as at least twice the background value, >0.05% after subtraction of background and at least 1,000 events. This criterion was established to minimize the possibility of error due to a low number of events when further subdividing these cells into the different memory subsets. Central memory T cells (T_CM_, CCR7+/CD45RO+), effector memory T cells (T_EM_, CCR7−/CD45RO+), and terminal effector T cells (T_TE_, CCR7−/CD45RO−) were identified. Within the CD45RO−CCR7+ cells naive T-cells (T_naive_, CCR7+/CD45RO−/CD95−) and stem-cell memory T-cells (T_SCM_, CCR7+/CD45RO−/CD95+) were gated. Additionally, PD-1 expression was studied on bulk and memory subpopulations. Based on the knowledge that HIV-specific CD8+ T-cell frequency decays significantly following cART initiation, an *in vitro* cell expansion protocol was implemented as reported previously by our group [21]. Phenotype and polyfunctionality were subsequently studied on expanded cells.

Due to limitations in sample availability, bulk CD8TC and CD4 TC responses at pre-cART samples were evaluated in 22 and 23 participants, respectively. In all participants except 1, replicates could be made, adding a total of 43 and 45 responses, respectively. Bulk CD8TC parameters at post-cART were evaluated in the 25 participants of the cohort, all of them in duplicate except for 3. This adds a total of 47 responses. The phenotypes of HIV-specific CD8TC responses were evaluated in 18 individuals pre-cART and in 23 individuals post-cART. When the quantity of sample allowed it, replicates were performed with different antigens in the stimulation (Nef and p24 peptide pools). Thus, a total of 35 responses were obtained pre-cART and 43 responses were obtained post-cART. CD8 TC polyfunctionality was evaluated in 24 and 23 pre-cART and post-cART samples, respectively. When possible, samples were tested in replicate against Nef and p24 peptide pools adding a total of 42 and 46 responses, respectively.

Additionally, antiviral activity of expanded CD8+ T cells was evaluated using the Viral Inhibition Assay (VIA) and the VITAL assay as described previously by our group [21]. Briefly, the VIA protocol was initially published by Sáez-Cirión et al [24] and it assesses the capacity of expanded CD8TC to inhibit viral replication in primary autologous CD4TCs, accounting for both cytolytic and non-cytolytic mechanisms. Here, target cells (consisting of autologous CD4TCs purified after PBMC treatment with CD3/8 bi-specific antibodies) were infected and co-cultured at 1:1 ratio with purified expanded Nef-specific or p24-specific CD8+ T cells (effectors). At day 7, p24 antigen was quantified in cell culture supernatants by ELISA. CD8TC anti-HIV suppressive capacity was calculated as the log_10_ of the percentage of p24 antigen loss when CD8TCs were present in the culture compared to infected CD4TC controls without effectors. On the other hand, the VITAL assay was adapted from the publication by Hermans et al [25], and it assesses direct HIV-specific cell-mediated cytotoxicity.

Briefly, CD4+ target cells (prepared as described above) were either labeled with CFSE or PKH-26 red fluorescent cell linker. CFSE-labeled cells were loaded with Nef or p24 peptides and then combined with PKH-26-labeled cells. Nef- or p24-specific effector cells were added at different target-to-effector (T:E) ratios (1:1, 1:5, 1:10, and 1:1 adjusted to proportion of specific CD8^+^ T-cell effectors). Following overnight incubation, cells were stained with Zombie Viability Kit and anti-CD3-PECy7, anti-CD4-PerCP, and anti-CD8-BV510 antibodies, and analyzed by flow cytometry. Adjusted survival was calculated as the mean percentage of CFSE^+^ events with added effector vs the condition with no effectors. Finally, the percentage of specific lysis was calculated using the equation % specific lysis= 100 - % adjusted survival. Further details can be found in Salido et al [21]. The VIA assay could be performed in 16 post-cART samples. In all cases, effector cells were prepared in parallel with Nef and p24 peptide pools resulting in 2 sets of effectors per sample that were tested separately. This resulted in 32 VIA values for the whole set of samples. Moreover, these 2 sets of effectors were obtained for the VITAL assay. However, this assay requires a larger number of cells and thus the number of samples tested and replicates were smaller: 1:1 relation: 15 samples, 30 responses; 1:5: 12 samples, 21 responses; 1:10: 10 samples, 15 responses.

### Quantitative real-time PCR for Cell-Associated (CA) HIV RNA and DNA:

Cell-associated (CA) HIV DNA (total and integrated) and RNA (unspliced [US]) and multiple spliced [MS]) were quantitated by real-time PCR on post-cART samples. Total HIV DNA PCR measures all forms of viral DNA present in the cell (integrated and non-integrated forms), while HIV-integrated DNA PCR amplifies integrated provirus exclusively. The CA US-RNA form represents the genomic RNA (result of genuine HIV transcription) but can also represent host-HIV read-through transcripts. However, the latter transcripts have been shown to contribute poorly to the bulk of HIV RNA. The CA MS-RNA transcripts are more closely related to advanced stages in the cycle of viral replication and production of infectious particles [26].

CD4^+^ T cells were isolated from frozen PBMCs using an immunomagnetic selection kit (BD Bioscience, USA) and only 90% purity samples (determined by flow cytometry) were assayed. DNA and RNA were extracted using a proteinase K lysis protocol or a commercial kit (PureLink RNA Mini Kit, Invitrogen, USA) respectively, then both were quantified and stored at −80°C until use. Total and integrated HIV DNA was quantitated following the highly sensitive real-time nested PCR-based assay developed by Chomont et al [27] with modifications. Briefly, cell lysates were used as input in a pre-PCR step in which the total or integrated HIV DNA was pre-amplified together with the CD3 gene (2 copies per cell) in the same reaction, in triplicate. Pre-amplified products were diluted and used as input in a second amplification reaction (HIV or CD3) in which each form was quantified by real-time PCR. The frequency of cells harboring each molecular form was calculated from the ratio of HIV copy number/2 x (CD3 copy number). For quantification of US and MS-RNA forms, Pasternak's protocol was used [28] with modifications. Briefly, a hemi-nested PCR was performed with 16 cycles of amplification followed by a second amplification round of quantitative real time PCR. Then US and MS-RNA copy numbers were standardized to cellular equivalents using a 18s RNA real time PCR (Invitrogen, USA). Each sample was assayed in quadruplicate and a control with no reverse transcriptase was used to reveal DNA contamination. Quantitative real time PCR assays were run for 40 cycles. For both HIV DNA and RNA PCRs, the LLODs were 1 copy per well. If there was amplification but it was not quantified (below the LLOD), an arbitrary value of 0.5 copies was assigned. If no amplification occurred at all, the value was set to 0 copies. Due to sample availability, these assays were performed in the following number of samples: Total HIV DNA: 17 samples, Integrated HIV DNA: 5 samples, CA HIV US-RNA: 18 samples, and CA HIV MS-RNA: 12 samples.

### Plasma Soluble factors:

The levels of CXCL10 (IP-10, BD Biosciences), soluble CD14 (sCD14) and soluble CD163 (sCD163) (Thermo Fisher Scientific) were measured in post-cART plasma samples. Due to sample availability, 9, 23, and 22 samples were used to evaluate IP-10, sCD14, and sCD163 respectively.

### Data analysis:

Data was expressed as median values with interquartile ranges (25% to 75%, IQ25-75) and analyzed by nonparametric methods using GraphPad Prism v7.0 software, unless otherwise stated. Correlation analyses were performed using the Spearman's rank test. In this case, *P*-values were adjusted for multiple comparisons using a false discovery rate (FDR) procedure, according to the Benjamini and Hochberg method, using the GraphPad Prism 7 software (Graph-Pad Software Inc., San Diego, CA, USA). Distribution analysis of CD4+ and CD8+ T-cell subsets in pre- and post-cART samples was performed with SPICE 6.0 software (https://niaid.github.io/spice/). All tests were considered significant when the *P*-value was <0.05. Adjusted *P*-values for correlation analyses were considered significant when <0.1.

## RESULTS

### Cohort description and experimental determinations:

Twenty-five participants from the *Grupo Argentino de Seroconversión* study group were selected. Samples were obtained immediately before cART initiation (pre-cART sample) and at a median of 15 months after cART initiation (post-cART samples). Paired pre- and post-cART samples were obtained for all participants but 3. A detailed description of participants is shown in Table 1. As expected, all participants had detectable viral load pre-cART and reached levels below the limit of detection post-cART.

Median CD8TC count significantly diminished while median CD4TC count and CD4/CD8 ratio significantly increased post-cART (Wilcoxon's test *P*<0.0001, *P*=0.0001, and *P*<0.0001, respectively).

At the pre-cART samples, the following determinations were performed: i) distribution of memory subpopulations at bulk CD4TC and CD8TC compartments; ii) HIV-specific CD8TC polyfunctionality and memory/effector differentiation profile; iii) PD-1 expression in bulk and memory/effector subpopulations of CD4TC, CD8TC, and HIV-specific cells. In post-cART samples, CA HIV DNA and RNA forms as well as plasma levels of IP-10, sCD14, and sCD163 were determined *ex vivo*. Also, the distribution of memory/effector subsets in bulk and HIV-specific CD8TC and HIV-specific CD8TC polyfunctionality were determined after cell expansion *in vitro* as performed previously [21]. Results for these determinations are shown in supplemental figures 1 and 2. Overall, it could be observed that the pre-cART distribution of naive and memory/effector T-cell subsets, both at the CD4TC and CD8TC compartments, behaved as expected according to previous publications [18, 29] (Supplemental Figure 1A). Since it is known that the frequency of HIV-specific T cells decays significantly following cART initiation, cells from post-cART samples were expanded as reported previously [21]. No immune parameter was evaluated directly *ex vivo* in post-cART samples. During the expansion process, CD8TC are preferentially expanded over CD4TCs. This could be due to suboptimal culture conditions for CD4TCs proliferation, out-competition by CD8TCs, underrepresentation of MHC-II-restricted compared to MHC-I-restricted peptides, or for other reasons. Thus, CD4TC were not evaluated at post-cART samples.

Both bulk and HIV-specific CD8TC post-cART also showed the anticipated phenotype [21] with a noticeable enrichment of effector memory (T_EM_) at the specific compartment (Supplemental Figure 1B). HIV-specific pre-cART CD8TC were dominated by monofunctional cells. The most frequent functions were CD107A/B mobilization (degranulation) and TNF-α production followed by IFN-γ, Mip1β (CCL4), and IL-2 production. On the other hand, the most frequent functions in expanded CD8TC from post-cART samples were IFN-γ, Mip1β (CCL4), and TNF-α production followed by degranulation and, to a lesser extent, IL-2 production. In this group, a significantly higher proportion of polyfunctional cells able to carry out multiple effector functions were found (permutation test *P*<0.0001, SPICE software; Supplemental Figure 1C). In line with this, expanded CD8TC were able to exert significant cytotoxic activity as measured by the VITAL assay, ie, specific direct lysis of HIV-peptide-loaded target CD4TCs by the autologous HIV-specific expanded CD8TC (Supplemental Figure 1D). Also, 12 out of 25 participants (52%) had detectable HIV-specific CD4TC responses pre-cART. Positive responses showed a median of 0.56% (IQR25-75=0.42%-0.86%, data not shown).

Pre-cART PD-1 showed a significant expression when analyzed in bulk CD4TC and CD8TC (Supplemental Figure 1E, left and right panel, respectively), especially in those individuals with delayed treatment initiation. When its expression was studied in the different subpopulations, it was concentrated within T_EM_ and central memory T cells (T_CM_). Regarding viral reservoir composition post-cART, total HIV DNA was more readily measured than integrated DNA, while US-RNA was more abundant than MS-RNA (Supplemental Figure 2A). Plus, US-RNA to total or integrated DNA ratios were calculated in order to account for transcriptional activity per infected cell; and MS- to US-RNA ratio to illustrate the stage in viral genome transcription, ie, lower MS/US-RNA reflects higher frequency of cells in the later stages of the viral replication cycle [26] (Supplemental Figure 2B). Finally, plasma levels of IP-10, sCD14, and sCD163 were measured. These molecules have been proposed as markers of persistent inflammation in HIV infection since their levels remain elevated despite years of cART, compared to healthy donors [30–37]. Both IP-10, sCD14, and sCD163 were detected in all the samples tested. Moreover, IP-10 and sCD14 medians were considerably elevated compared to values corresponding to healthy donors found in the bibliography (Supplemental Figure 2C) [30–34, 38–41].

### Correlation analysis showed a relationship between pre- and post-cART CD8TC response quality (preserved distribution of memory subsets, functionality, and lower PD-1 expression), while CD4TC phenotype (distribution of memory subsets and PD-1 expression) pre-cART was better associated with the level of HIV persistence and inflammation post-cART.

As a first approach on how pre-cART CD8TC and CD4TC phenotype and function relate to the parameters evaluated post-cART, correlation analyses were performed and heat-maps were prepared to better visualize results (Supplemental Figure 3). The rows and columns correspond to determinations performed at the pre-cART and post-cART samples, respectively. Also, the estimated time of infection to sampling and the pre-cART VL value were included as row variables. In this manner, we were able to identify patterns of consistent correlations that provide support to the ideas presented in our hypothesis:

### i) The phenotype of CD8TC pre-cART influences the functionality of CD8TC post-cART:

**Figure 1:**
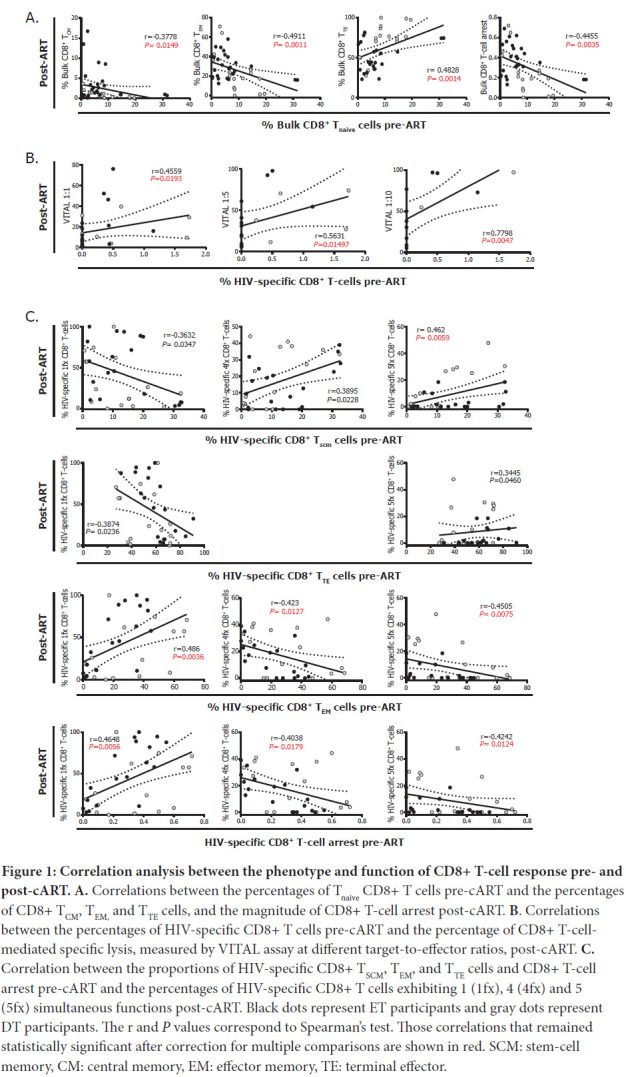
**Correlation analysis between the phenotype and function of CD8+ T-cell response pre- and post-cART. A.** Correlations between the percentages of T_naive_ CD8+ T cells pre-cART and the percentages of CD8+ T_CM_, T_EM,_ and T_TE_ cells, and the magnitude of CD8+ T-cell arrest post-cART. **B**. Correlations between the percentages of HIV-specific CD8+ T cells pre-cART and the percentage of CD8+ T-cell-mediated specific lysis, measured by VITAL assay at different target-to-effector ratios, post-cART. **C.** Correlation between the proportions of HIV-specific CD8+ T_SCM_, T_EM_, and T_TE_ cells and CD8+ T-cell arrest pre-cART and the percentages of HIV-specific CD8+ T cells exhibiting 1 (1fx), 4 (4fx) and 5 (5fx) simultaneous functions post-cART. Black dots represent ET participants and gray dots represent DT participants. The r and *P* values correspond to Spearman's test. Those correlations that remained statistically significant after correction for multiple comparisons are shown in red. SCM: stem-cell memory, CM: central memory, EM: effector memory, TE: terminal effector.

First, we observed that the percentages of pre-cART bulk CD8^+^ T_naive_ cells correlated inversely with the proportions of post-cART bulk CD8^+^ T_CM_, T_EM_ cells and with the levels of CD8^+^ T-cell arrest (calculated as the ratio T_EM_/[T_EM_+T_TE_] as described previously [18]), while it correlated directly with the proportions of bulk CD8^+^ T_TE_ cells (Figure 1A). The percentage of HIV-specific CD8TC pre-ART evaluated *ex vivo* by flow cytometry correlated directly with the capacity of expanded CD8TC from post-cART samples to mediate direct cytolytic antiviral function measured by the VITAL assay (Figure 1B). In addition, the phenotype of the HIV-specific CD8^+^ T cells pre-cART was related to cell functionality post-cART: higher percentages of CD8^+^ stem-cell memory T cells (T_SCM_) and T_TE_ cells correlated inversely with proportions of monofunctional HIV-specific CD8^+^ T cells post-cART and correlated directly with higher proportions of polyfunctional (4- and 5-function cells for T_SCM_ and 5-function cells for T_TE_) post-cART. Conversely, higher percentages of CD8^+^ T_EM_ cells and higher arrest correlated directly with higher frequencies of monofunctional cells (Figure 1C) and correlated inversely with frequencies of polyfunctional HIV-specific CD8^+^ T cells post-cART.

### ii) An exhausted phenotype on CD8^+^ T cells pre-cART also relates to HIV persistence and inflammation post-cART:

**Figure 2:**
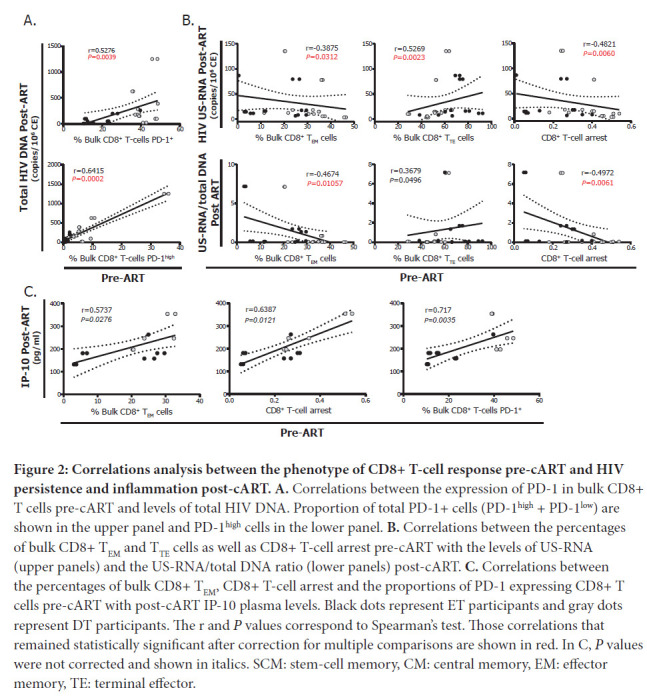
**Correlations analysis between the phenotype of CD8+ T-cell response pre-cART and HIV persistence and inflammation post-cART. A.** Correlations between the expression of PD-1 in bulk CD8+ T cells pre-cART and levels of total HIV DNA. Proportion of total PD-1+ cells (PD-1^high^ + PD-1^low^) are shown in the upper panel and PD-1^high^ cells in the lower panel. **B.** Correlations between the percentages of bulk CD8+ T_EM_ and T_TE_ cells as well as CD8+ T-cell arrest pre-cART with the levels of US-RNA (upper panels) and the US-RNA/total DNA ratio (lower panels) post-cART. **C.** Correlations between the percentages of bulk CD8+ T_EM_, CD8+ T-cell arrest and the proportions of PD-1 expressing CD8+ T cells pre-cART with post-cART IP-10 plasma levels. Black dots represent ET participants and gray dots represent DT participants. The r and *P* values correspond to Spearman's test. Those correlations that remained statistically significant after correction for multiple comparisons are shown in red. In C, *P* values were not corrected and shown in italics. SCM: stem-cell memory, CM: central memory, EM: effector memory, TE: terminal effector.

The percentages of CD8^+^ T cells expressing PD-1 and PD-1^high^ pre-cART were correlated directly with higher levels of total HIV DNA (Figure 2A). Higher percentages of CD8^+^ T_EM_ cells, lower percentages of CD8^+^ T_TE_ cells and higher CD8^+^ T-cell arrest were related to lower levels of HIV US-RNA and lower US-RNA/total DNA ratios post-cART (Figure 2B). Finally, frequencies of CD8^+^ T_EM_ cells, CD8^+^ T–cell arrest, and the frequency of CD8^+^ T-cells expressing PD-1 correlated directly with the levels of plasma IP-10 post-cART (Figure 2C).

### iii) Exhaustion and differentiation of CD4^+^ T cells pre-cART determine the composition of the HIV reservoir post-cART and also the level of inflammation:

**Figure 3:**
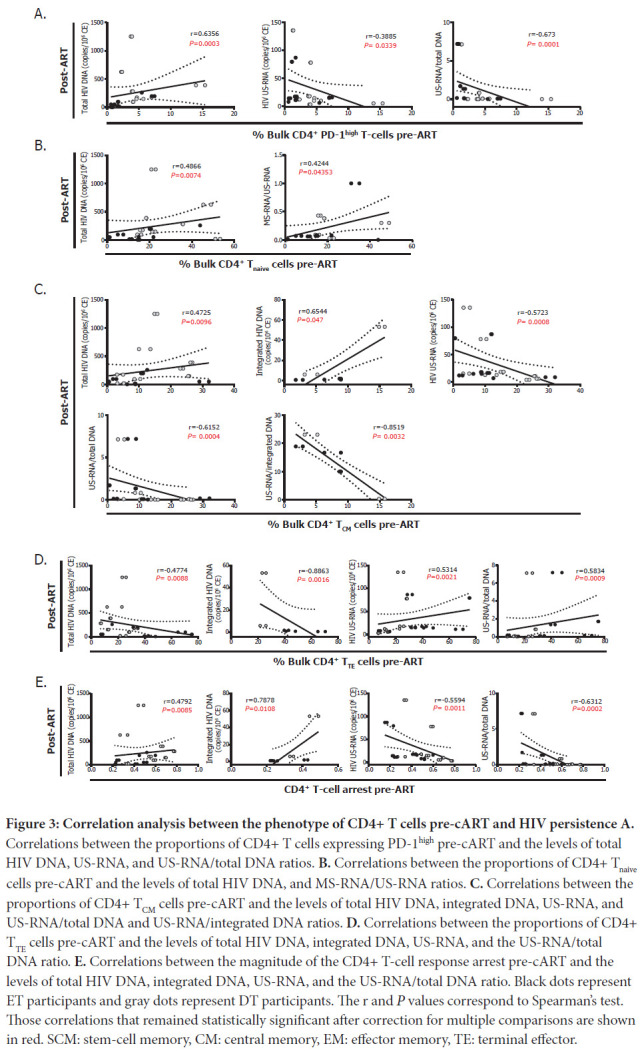
**Correlation analysis between the phenotype of CD4+ T cells pre-cART and HIV persistence A.** Correlations between the proportions of CD4+ T cells expressing PD-1^high^ pre-cART and the levels of total HIV DNA, US-RNA, and US-RNA/total DNA ratios. **B.** Correlations between the proportions of CD4+ T_naive_ cells pre-cART and the levels of total HIV DNA, and MS-RNA/US-RNA ratios. **C.** Correlations between the proportions of CD4+ T_CM_ cells pre-cART and the levels of total HIV DNA, integrated DNA, US-RNA, and US-RNA/total DNA and US-RNA/integrated DNA ratios. **D.** Correlations between the proportions of CD4+ T_TE_ cells pre-cART and the levels of total HIV DNA, integrated DNA, US-RNA, and the US-RNA/total DNA ratio. **E.** Correlations between the magnitude of the CD4+ T-cell response arrest pre-cART and the levels of total HIV DNA, integrated DNA, US-RNA, and the US-RNA/total DNA ratio. Black dots represent ET participants and gray dots represent DT participants. The r and *P* values correspond to Spearman's test. Those correlations that remained statistically significant after correction for multiple comparisons are shown in red. SCM: stem-cell memory, CM: central memory, EM: effector memory, TE: terminal effector.

The frequency of PD-1^high^ CD4^+^ T cells pre-cART correlated directly with the levels of total HIV DNA and inversely with US-RNA and the US-RNA/total DNA ratio post-cART (Figure 3A). Pre-cART percentages of CD4^+^ T_naive_ cells correlated directly with the levels of total HIV DNA, and MS-RNA/US-RNA ratios post-cART (Figure 3B). Similarly, pre-cART percentages of CD4^+^ T_CM_ cells correlated directly with the levels of total and integrated HIV DNA, and inversely with the levels of HIV US-RNA as wells as US-RNA/total DNA and US-RNA/integrated DNA ratios (Figure 3C). Finally, the percentages of terminally differentiated CD4^+^ T cells pre-cART correlated inversely with the total and integrated HIV DNA, and directly with the levels of US-RNA and the US-RNA/total DNA ratio (Figure 3D). Exactly opposite correlations were found with the magnitude of CD4^+^ T-cell differentiation arrest (Figure 3E). These results prompted us to study the relation of PD-1 expression within each of these CD4^+^ T-cell subpopulations and HIV persistence.

**Figure 4:**
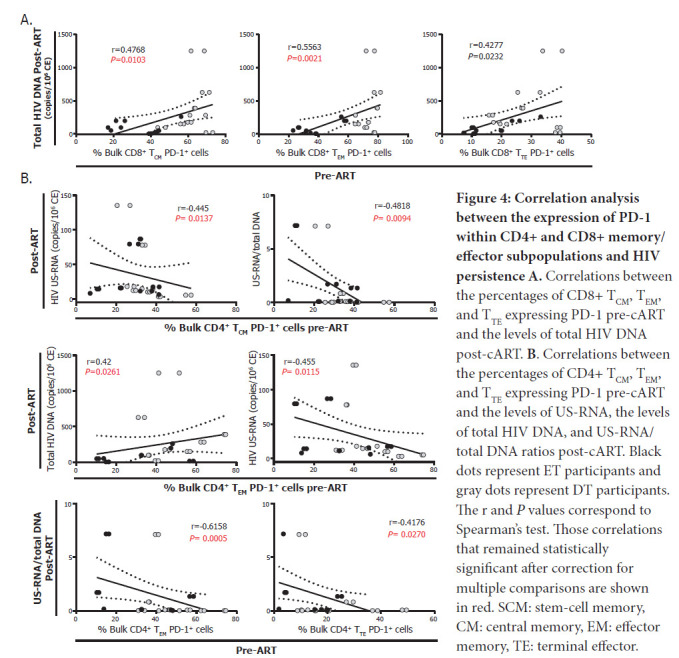
**Correlation analysis between the expression of PD-1 within CD4+ and CD8+ memory/effector subpopulations and HIV persistence A.** Correlations between the percentages of CD8+ T_CM_, T_EM_, and T_TE_ expressing PD-1 pre-cART and the levels of total HIV DNA post-cART. **B**. Correlations between the percentages of CD4+ T_CM_, T_EM_, and T_TE_ expressing PD-1 pre-cART and the levels of US-RNA, the levels of total HIV DNA, and US-RNA/total DNA ratios post-cART. Black dots represent ET participants and gray dots represent DT participants. The r and *P* values correspond to Spearman's test. Those correlations that remained statistically significant after correction for multiple comparisons are shown in red. SCM: stem-cell memory, CM: central memory, EM: effector memory, TE: terminal effector.

First, higher proportions of CD8^+^ T_CM_, T_EM_ and T_TE_ cells expressing PD-1 pre-cART were correlated with higher levels of total HIV DNA post-cART (Figure 4A), suggesting an association between an exhausted CD8^+^ T-cell response and a higher HIV reservoir post-cART. On the other hand, higher proportions of CD4^+^ T_CM_ PD-1^+^ pre-cART correlated with lower levels of US-RNA and US-RNA/total DNA ratios post-cART while higher proportions of CD4^+^ T_EM_ PD-1^+^ pre-cART correlated with higher levels of total DNA, lower US-RNA levels, and lower US-RNA/total DNA ratios post-cART. In turn, proportions of CD4^+^ T_TE_ PD-1^+^ pre-cART correlated inversely with US-RNA/total DNA ratios (Figure 4B). Overall, these results indicate that HIV may persist in diverse CD4^+^ T-cell subsets with different efficiency. Pre-cART expression of PD-1 on CD4^+^ T cells seems to be a marker of lower HIV transcriptional activity, particularly in T_CM_ and T_EM_ cells.

**Figure 5:**
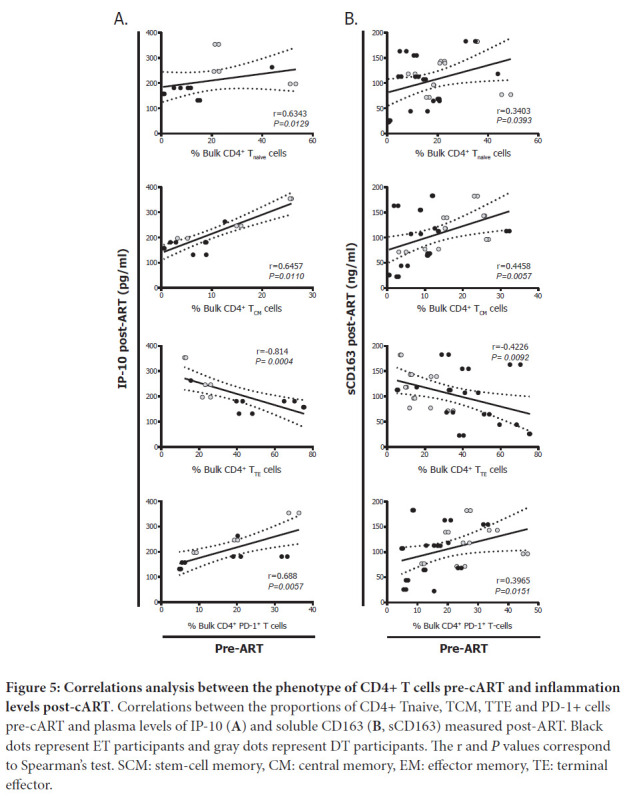
**Correlations analysis between the phenotype of CD4+ T cells pre-cART and inflammation levels post-cART**. Correlations between the proportions of CD4+ Tnaive, TCM, TTE and PD-1+ cells pre-cART and plasma levels of IP-10 (**A**) and soluble CD163 (**B**, sCD163) measured post-ART. Black dots represent ET participants and gray dots represent DT participants. The r and *P* values correspond to Spearman's test. SCM: stem-cell memory, CM: central memory, EM: effector memory, TE: terminal effector.

Finally, percentages of pre-cART CD4^+^ T_naive_ and T_CM_ cells correlated directly with plasma levels of IP-10 and sCD163 post-cART while proportions of pre-cART CD4^+^ T_TE_ cells showed inverse correlations (Figure 5). In turn, percentages of pre-cART CD4^+^ PD-1^+^ T cells correlated directly with both markers.

## DISCUSSION

**Figure 6:**
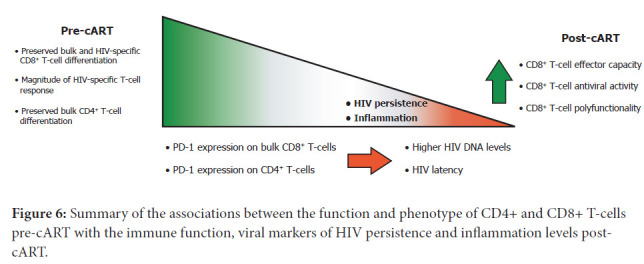
Summary of the associations between the function and phenotype of CD4+ and CD8+ T-cells pre-cART with the immune function, viral markers of HIV persistence and inflammation levels post-cART.

HIV-specific CD8^+^ T-cell responses are believed to be a very important factor in achieving sustained long-term viral control as demonstrated in non-human primate models and elite controllers [16]. Since responses elicited in most PLWHA are sub-effective, developing strategies to promote de novo responses or to modulate existing memory immunity in order to resemble the characteristics found in HIV controllers may pave the way to cure the infection. Here, we extended previous studies regarding the association of function and phenotype of CD4^+^ and CD8^+^ T cells pre-cART with the immune function and viral markers that persist after cART instauration. It was found that i) the phenotype of CD8^+^ T cells pre-cART as well as the magnitude and pheno-type of HIV-specific response might be associated with the phenotype and functionality of CD8^+^ T cells post-cART; ii) the phenotype of the CD8^+^ T cells pre-cART correlated with markers of HIV persistence and inflammation post-cART; iii) exhaustion and differentiation of CD4^+^ T cells pre-cART were associated with the composition of the HIV reservoir post-cART and the level of inflammation (Figure 6).

During the natural course of infection, HIV-specific CD8^+^ T cells play a central role in the control of viral replication, particularly during acute infection [16, 42, 43]. Bulk and HIV-specific CD8^+^ T cells suffer early alterations in their frequency, differentiation, activation, and functionality that are progressively accentuated, as antigen stimulation persists in untreated infection [17, 18, 44, 45]. As PLWHA initiate cART, some of these alterations revert but CD8 T-cell optimal function is not fully restored [42, 46–54]. As more CD8^+^ T-cell-based vaccine or CD8^+^ T-cell dependent cure strategies are being proposed and developed, it is critical to understand and identify the limitations and pitfalls of the immune response that persists in people receiving cART, and to generate tools in order to boost its antiviral function. Here, we show that the magnitude of the damage reached pre-cART associates with the level of dysfunction that remains when receiving cART. This is illustrated by correlations found between percentages of bulk CD8^+^ T_naive_ cells pre-cART and the level of CD8TC arrest post-cART (Figure 1A), the level of CD8TC arrest in the HIV-specific CD8^+^ T-cell compartment pre-cART and decreased functionality post-cART (Figure 1B-C), and the level of CD8TC arrest and exhaustion of the bulk compartment pre-cART with the levels of inflammation when receiving cART (Figure 2C). It is worth noting that the level of CD8TC arrest as calculated here is an indicator of maturation blockade and skewed differentiation in the T-cell compartment in HIV infection, and it has been correlated with markers of disease progression [18, 46, 55–57]. Thus, these CD8^+^ T-cell parameters, evaluated immediately before cART initiation, could serve as markers of CD8^+^ T-cell functionality post-cART, could determine if an individual is eligible for a given intervention, or could be useful to predict the success of a CD8^+^ T-cell-based strategy or even the need of tailored interventions. In addition, we report that percentages of exhausted bulk CD8^+^ T cells as well as the level of differentiation arrest in this compartment correlated with the size of the HIV DNA reservoir during cART and the activity of this reservoir, respectively (Figure 2A-B). This recapitulates and extends our previous finding [20] where similar results were obtained in an independent cohort. Two hypotheses arise; it could be first postulated that an early preservation of the CD8^+^ T-cell compartment limits viral spreading resulting in a smaller reservoir. It is worth noting that the correlations obtained referred to the bulk compartment, not the HIV-specific compartment. This could be illustrating the global alteration that this cell population undergoes in the context of HIV infection. On the other hand, it is known that CD8^+^ T cells have a role in controlling viral replication during cART although it is neither complete nor efficient since VL inevitably re-emerges upon cART interruption [12, 16, 20, 29, 58–61]. Thus, pre-cART CD8^+^ T-cell response quality might be standing as a surrogate marker for CD8^+^ T-cell response quality during cART in these correlations. Recent advances indicate that viral proteins were found to be expressed from latently infected CD4^+^ T cells, even from those harboring defective HIV proviruses, and that these cells are subjected to immune surveillance [62, 63]. Very recently, Stevenson et al [64] showed that long-term CD8^+^ T-cell responses during cART are maintained by viral protein-mediated cell triggering. This indicates that CD8+ T-cell response during cART contributes to the dynamics of reservoir maintenance, and warrants more research to understand the molecular biology of HIV persistence during cART and its interaction with the CD8^+^ T-cell response in order to expand cure and vaccine research.

The population of CD4^+^ T cells is very heterogeneous and susceptibility to HIV infection and replication activity depends on their differentiation, activation status, functionality, phenotype, and even antigen specificity, among other factors [65]. In treated participants, frequency of HIV persistence varies across different memory subsets. While CD4^+^ T_naive_ cells and T_SCM_ are known to contribute to viral persistence, T_CM_ and T_EM_ are the key contributors to the viral reservoir based not only on their own capacity to self-renew and proliferate but also because they show the highest frequency of infected cells [66, 67] and the highest frequencies of intact and inducible proviruses [68–72], respectively. Here, correlations were observed between the proportions of different CD4^+^ T-cell subsets pre-cART and either the magnitude of the HIV reservoir measured by total or integrated DNA or the transcriptional activity of that reservoir measured as levels of US-RNA or MS-RNA or HIV RNA/DNA ratios. Levels of HIV DNA post-cART (total and/or integrated) were associated with higher proportions of CD4^+^ T_Naive_ cells, CD4^+^ T_CM_ cells, higher CD4^+^ T-cell arrest, and lower proportions of CD4^+^ T_TE_ cells pre-cART. However, exactly opposite correlations were found when measuring the transcriptional activity (RNA forms) of the reservoir (Figure 3). This is consistent with the idea that CD4^+^ T_CM_ cells contribute highly to the pool of HIV-infected cells that persist after cART, but that they harbor virus which rather keeps in a transcriptionally silent state. Then, HIV transcription activity augments as memory cells transition to an activated effector phenotype [65]. In addition to the cellular differentiation state, the expression and coexpression of certain immune checkpoint molecules on CD4^+^ T-cells (PD-1, TIGIT, LAG-3, CTLA4, among others) have been associated with different states of HIV persistence [73–76].

In particular, we have previously shown that the magnitude of PD-1 expression both at the CD8^+^ and CD4^+^ T-cell compartments, evaluated early after infection, was associated with the levels of subsequent viral persistence on cART [20]. Here, we show that although pre-cART expression of PD-1 on CD4^+^ T-cells associates directly with the frequency of infected cells on cART, higher frequencies of CD4^+^ PD-1^+^ T_CM_, T_EM_ and T_TE_ cells correlated with lower HIV transcription during cART measured as the level of US-RNA or US-RNA/total DNA ratio. This finding is in line with previous evidence suggesting that PD-1 (and other immune checkpoints) are markers of transcriptionally-silent HIV-infected cells during cART [73, 74, 76]. Moreover, blockade of immune checkpoints (more specifically PD-1) was shown to rescue HIV transcription in these cells, and that it could even be proposed as a latency reversal agent [77]. So far, most cure interventions have been proposed to start once PLWHA are receiving suppressive cART. However, it may also be possible that targeting PD-1 at cART initiation could also be beneficial, leading to a smaller HIV reservoir post-cART by reversing HIV latency and increasing CD8+ T-cell function against infected cells earlier.

This is a descriptive study seeking correlations between immune and viral parameters associated with HIV infection. We performed a comprehensive study where multiple immunological and virological parameters were evaluated in paired pre- and post-cART samples. Although our approach is only descriptive, many of the associations that were raised in the analyses are in line with findings from studies involving mechanistical or interventional approaches. This provides reciprocal support to the findings and enhances the evidence in the field. As discussed above, several pre-cART parameters identified here have the potential to act as biomarkers of post-cART immune function and viral persistence, and, hypothetically, could also serve to predict the outcome of an intervention or even indicate whether the intervention is adequate for a given person. Furthermore, it is possible to postulate that the moment of cART initiation may also represent a moment of opportunity to intervene.

This study has several limitations. First, limited sample availability resulted in discrepancies in the number of parameters evaluated in each sample. The second limitation was the need to use a strong and prolonged stimulation protocol for T-cell expansion in samples obtained during cART. Although it is a useful tool to evidence the HIV-specific response post-cART, it could also introduce modifications into the cell phenotype and function. Third, cell phenotype was only studied by means of memory differentiation markers and PD-1 expression. Deeper analysis including other immune checkpoint molecules, co-stimulatory molecules, and survival markers could provide additional and relevant information. Similarly, inclusion of other functional markers such as the production of perforins and granzymes, which have been already described as key components of CD8^+^ T-cell antiviral function, were not measured.

Fourth, pre-cART and post-cART samples are not homogeneous in terms of time from infection and time receiving cART, respectively. Moreover, DT and ET groups differ significantly in time from infection to sampling and pre-cART VL values. Indeed, it is well known that these 2 variables play a significant role in immune function preservation, inflammation, immune-mediated damage, and composition and size of the viral reservoir. In this study, global associations between pre- and post-cART parameters are described without pretending to imply a cause-effect relationship nor suggest a direct underlying mechanism that could account for such associations. Whether the time from infection to sampling or the pre-cART VL values influence these associations could not be discounted. A proper study with an adequate sample size should be designed to rule out this possibility and exclude confounders. On the other hand, immune profiling of CD4TC was not performed in post-ART samples due to technical constraints and lack of enough biological material. Certainly, this could have represented an added value to the study and deserves to be analyzed in a subsequent study. Finally, PCR-based assays aimed at quantifying HIV DNA or RNA have been extensively used to study the viral reservoir in PLWHA receiving cART. However, these techniques fail to discriminate between intact vs defective or inducible vs latent proviruses. This may overestimate the size of the replication-competent virus pool, and the results may not necessarily correlate with the dynamics of the truly inducible, replication-competent viral reservoir. Our results should be extended by including other measurements such as the qVOA, TILDA (Tat/rev Induced Limiting Dilution Assay), and IPDA (intact proviral DNA assay) assays [78].

Overall, combined and parallel analysis of CD8^+^ and CD4^+^ T-cell quality before cART together with immune and viral factors during cART represent an important progress in order to understand HIV persistence. Boosting and/or modulation of CD8^+^ T-cell responses in individuals receiving cART is postulated as essential in HIV cure strategies. Here, we observed that pre-cART quality in CD8^+^ T-cell response could predict the level of functionality of CD8^+^ T-cell response during cART. Similarly, proportions of the different CD4^+^ T-cell memory/effector subsets and the expression of exhaustion markers within each subset could act as determinants not only of the level but also of the activity of the viral reservoir. Advances made in the HIV persistence field over the last few years hold promise for the development of HIV functional cure strategies. Within these strategies, the immune environment will be a key determinant of success. This work provides data to help understand and identify parameters that could be used as markers for subsequent interventions.

## SUPPLEMENTARY DATA

Supplementary materials are available at the Pathogens and Immunity website. Supplementary data may be provided by the authors to benefit the reader. Supplementary data are not copyedited and are the sole responsibility of the authors. Questions or comments related to supplementary materials should be addressed to the corresponding author.
