# Supplementary material for: Pre-cART Immune Parameters in People Living With HIV Might Help Predict CD8+ T-Cell Characteristics, Inflammation Levels, and Reservoir Composition After Effective cART
Source: Pathog Immun. 2021 Oct 1;6(2):60–89. doi: 10.20411/pai.v6i2.447 (PMC8714178; doi:10.20411/pai.v6i2.447)
Supplement: Supplemental Figures 1-3 [file pai-6-060-s01.pdf]

PATHOGENS AND IMMUNITY
IN ASSOCIATION WITH CASE WESTERN RESERVE UNIVERSITY AND THE RICHARD J. FASENMYER FOUNDATION

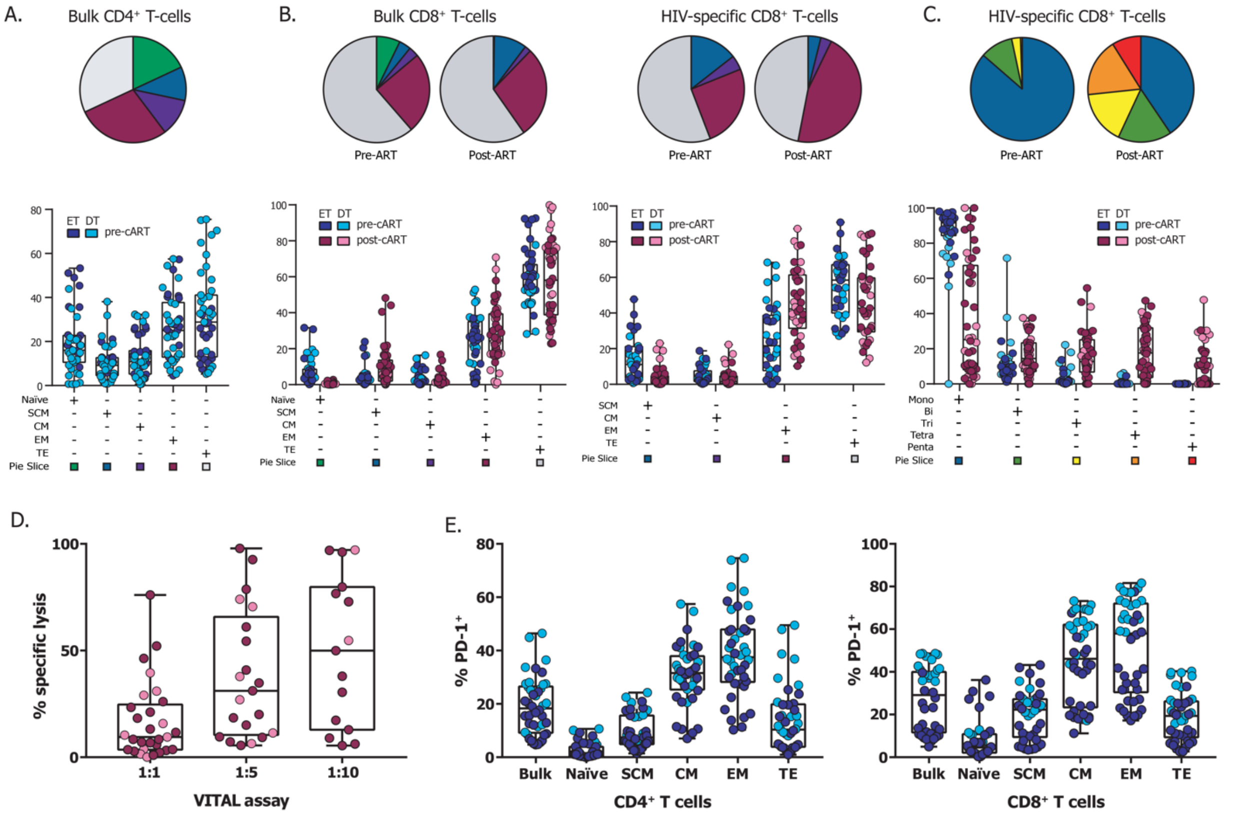

Supplemental Figure 1: Phenotype and functionality of CD4+ and CD8+ T-cell responses in HIV+ participants. A. Distribution of memory/effector subsets in bulk CD4+ T-cells pre-cART performed with SPICE 6.0 software (https://niaid.github.io/spice/). **B.** Distribution of memory/effector subsets in bulk (left panel) and HIV-specific (right panel) CD8+ T-cells at pre- and post (expanded)-cART samples. **C**. Distribution of mono-(blue), bi-(green), tri-(yellow), tetra-(orange), and penta-functional (red) cells within HIV specific CD8+ T-cells, pre- and post-cART. **D.** Percentage of specific lysis exerted by HIV-specific CD8+ T-cells in post-cART expanded samples, as determined by the VITAL assay. Results from target:effector 1:1, 1:5 and 1:10 ratios are shown. **E.** Pre-cART PD-1 expression in bulk and memory/effector subsets within the CD4+ (left panel) and CD8+ (right panel) T-cell compartments. In A-C, the color code shown at the bottom of the bar graph mirrors the one shown in the pies. Boxes extend from IQR25 to IQR75, whiskers extend from min to max and the horizontal bar within boxes represent median values. Within each data subset, dark dots (blue, dark red) represent ET subjects and light dots (light blue, pink,) represent DT subjects. SCM: stem-cell memory, CM: central memory, EM: effector memory, TE: terminal effector.

Salido J, Czernikier A, Trifone C, Polo ML, Figueroa MI, Urioste A, Cahn P, Sued O, Salomon H, Laufer N, Ghiglione Y, Turk G. Pre-cART Immune Parameters in People Living with HIV Might Help Predict CD8+ T-Cell Characteristics, Inflammation Levels and Reservoir Composition after effective cART. *Pathogens and Immunity*. 2021;6(2): 60-89. doi: 10.20411/pai.v6i2.447

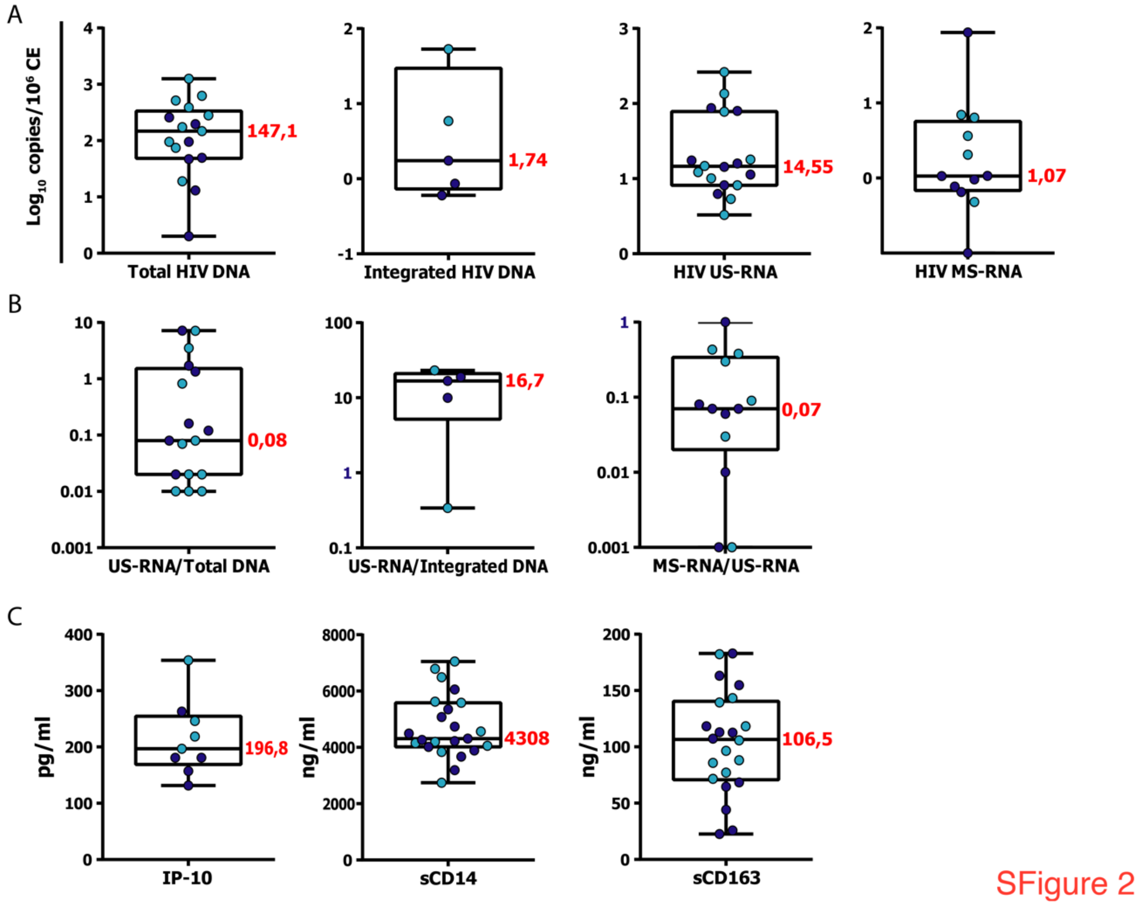

Supplemental Figure 2: Post-cART levels of HIV genomic forms and inflammation markers. A. Cell-associated (CA) total HIV DNA, Integrated HIV DNA, unspliced (US)-RNA and multiple spliced (MS)-RNA levels measured on samples obtained from participants on-cART. Viral DNA and RNA copies were calculated relative to 10^6 cell equivalents (CE). **B.** US-RNA vs. total DNA, US-RNA vs. integrated DNA and MS/US-RNA ratios. **C.** Post-cART plasma levels of IP-10 (CXCL-10), soluble CD14 (sCD14) and soluble CD163 (sCD163). Blue dots represent ET subjects and light-blue dots represent DT subjects. Boxes extend from IQR25 to IQR75, whiskers extend from min to max and the horizontal bar within boxes represent median values. Actual median values are shown in red next to each graph.

Salido J, Czernikier A, Trifone C, Polo ML, Figueroa MI, Urioste A, Cahn P, Sued O, Salomon H, Laufer N, Ghiglione Y, Turk G. Pre-cART Immune Parameters in People Living with HIV Might Help Predict CD8+ T-Cell Characteristics, Inflammation Levels and Reservoir Composition after effective cART. *Pathogens and Immunity*. 2021;6(2): 60-89. doi: 10.20411/pai.v6i2.447

PATHOGENS AND IMMUNITY
In Association with Case Western Reserve University and The Richard J. Fasenmyer Foundation

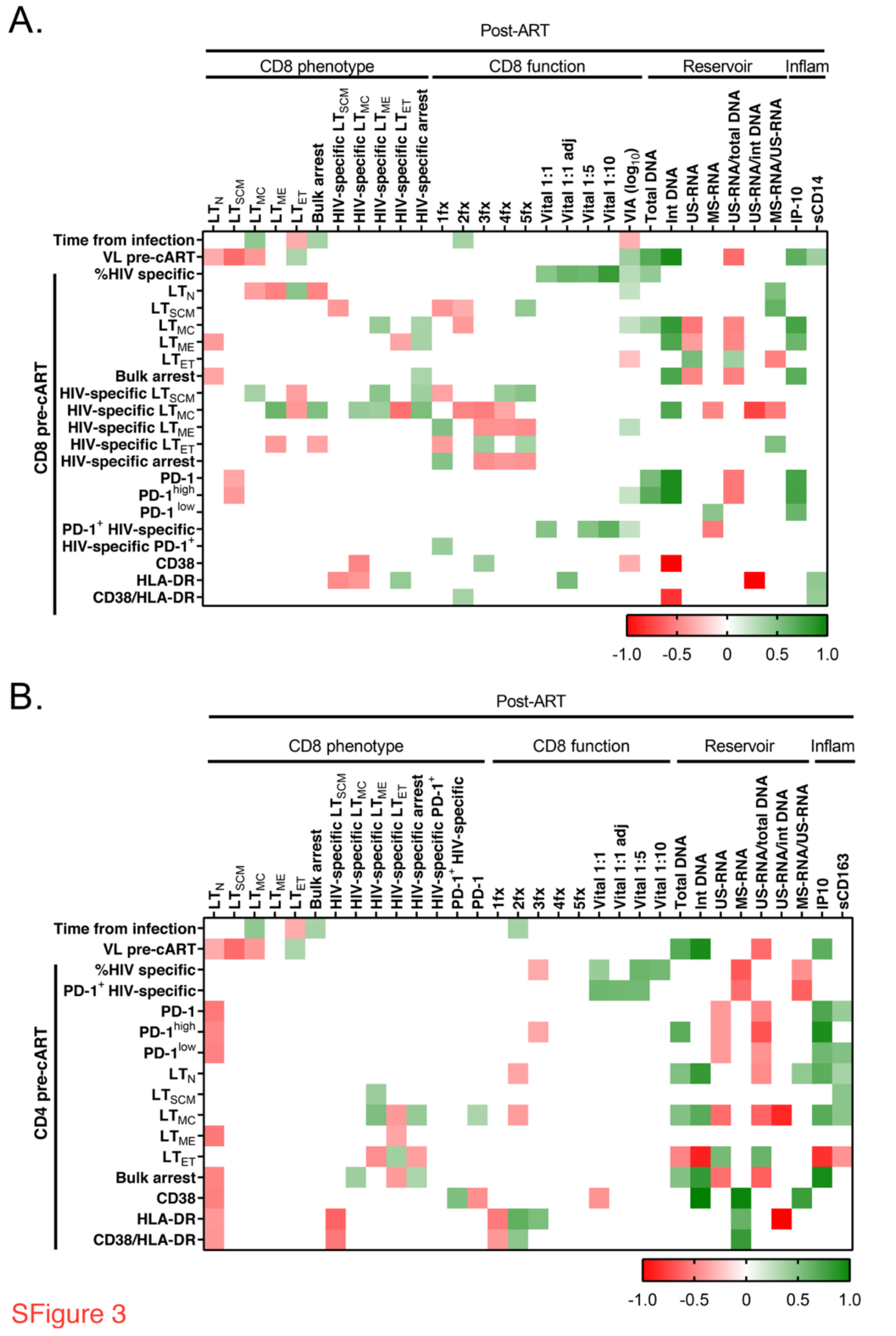

SFigure 3

Supplemental Figure 3: Associations between the immune profile of CD4+ and CD8+ T-cells pre-cART, and phenotype and function of CD8+ T-cells, HIV persistence and inflammation post-cART. Heat-maps analysis shows r values resulting from pairwise Spearman´s correlations between pre-cART parameters (rows) and post-ART parameters (columns) for CD4+ (A) and CD8+ T-cells (B).The colors denote both the correlation direction and strength of association, ranging from -1 (red) to 1 (green). Statistically non -significant (p>0.05) r values are shown in white.

Salido J, Czernikier A, Trifone C, Polo ML, Figueroa MI, Urioste A, Cahn P, Sued O, Salomon H, Laufer N, Ghiglione Y, Turk G. Pre-cART Immune Parameters in People Living with HIV Might Help Predict CD8+ T-Cell Characteristics, Inflammation Levels and Reservoir Composition after effective cART. *Pathogens and Immunity*. 2021;6(2): 60-89. doi: 10.20411/pai.v6i2.447
